# ADMA as a possible marker of endothelial damage. A study in young asymptomatic patients with cerebral small vessel disease

**DOI:** 10.1038/s41598-019-50778-w

**Published:** 2019-10-02

**Authors:** Francesco Janes, Adriana Cifù, Maria Elena Pessa, Rossana Domenis, Gian Luigi Gigli, Nova Sanvilli, Annacarmen Nilo, Riccardo Garbo, Francesco Curcio, Roberta Giacomello, Martina Fabris, Mariarosaria Valente

**Affiliations:** 1 Department of Neuroscience, S. Maria della Misericordia University Hospital, Udine, Italy; 2 Department of Laboratory Medicine, S. Maria della Misericordia University Hospital, Udine, Italy; 3 Department of Medical Area (DAME), University of Udine, Udine, Italy

**Keywords:** Stroke, Diagnostic markers, Stroke

## Abstract

Sporadic small vessel disease (SVD) has high prevalence in aging population and stroke patients, but also in younger asymptomatic subjects. In this last group it can represents a prelude to stroke and cognitive impairment. Still nowadays, its pathogenesis is unclear. 35 consecutive patients with SVD at brain MRI and 35 age- and sex-matched controls, between January 2016 and February 2018, underwent an extended screening for thrombophilia, autoimmunity and evaluated levels of blood markers of inflammation and endothelial activation. Asymmetric DiMethyl Arginine (ADMA) levels proved higher in patients (70.44 ± 36.25 ng/ml vs. 46.58 ± 30.67 ng/ml; p = 0.004), also after controlling for confounding factors. ADMA levels showed positive correlation with Fazekas score (r = 0.304; p = 0.01). ROC curve analysis showed a moderate accuracy in discriminating patients and controls (AUC = 0.70; CI 0.57–0.82; p = 0.004): a cut-off of 46 ng/ml is associated with 80% sensitivity, but limited (54%) specificity. Higher ADMA levels characterize selected subjects with sporadic SVD, asymptomatic for vascular diseases and without latent inflammatory conditions or coagulopathy. This reinforces the hypothesis of the key role of endothelial dysfunction in SVD. Further studies should explore the cause-effect relationship between ADMA pathway and SVD.

## Introduction

Small vessel disease (SVD) refers to a group of pathological processes with various aetiologies involving perforating cerebral arterioles, venules and capillaries, resulting in damage of cerebral white and deep grey matter. It includes lacunas, recent small subcortical infarcts, white-matter hyperintensities (WMH), perivascular spaces (PVS), micro bleeds (MB) and brain atrophy^1^. Several Authors refer to the most common form as the so-called “age-related SVD”, that is considered to be related to physiological ageing and classical vascular risk factors^2^.

Although in early phases SVD could be asymptomatic, the LADIS (Leukoaraiosis And DIsability Study) study group demonstrated its large health burden: severe clinical pictures more than double the risk of transition from an autonomous to a dependent status after 3 years of follow-up^3,4^; SVD is a premorbid condition in about the 20% of all strokes and about 45% of dementias; in older people it is related to gait disorders, mild cognitive impairment and depressive symptoms^4^. The increasing clinical use of cerebral MRI, even in the instrumental evaluation of benign neurologic complaints (headache, dizziness and vertigo, nonspecific cognitive or mood disorders), has led to apparently incidental finding of SVD also in young people, without classical vascular risk factors, familial history of vascular disease or suffering from chronic inflammatory disorders.

In order to clarify SVD pathogenesis, numerous biomarkers of coagulation, inflammation and endothelial dysfunction have been studied, but often with discordant results. Thus, different pathogenetic hypothesis are still under debate^5^. This might derive from the clinical heterogeneity of samples and from differences in the selection of biomarkers that were the object of the different studies in this field. In fact, several studies examined selectively biomarkers of fibrinolysis, coagulation, inflammation or nitric oxide pathway and only few evaluated all of them in a highly selected population^6^.

In order to avoid these selection limitations, we tried to investigate different pathways implicated in SVD pathogenesis (inflammation, coagulation and endothelial dysfunction) in a selected homogeneous population of patients with WMH, who did not present significant vascular risk factors or other neurological disorders and were relatively young in the context of cerebrovascular disease epidemiology^7–9^. Among the different biomarkers that have been investigated, only Asymmetric DiMethyl Arginine (ADMA), an endogenous inhibitor of endothelial nitric oxide synthase that has been linked in several studies to endothelial damage and cardiovascular diseases^10^, displayed significantly higher levels in patients than in controls.

## Results

We enrolled 35 patients and 35 age and sex-matched controls. Baseline characteristics of the all sample are summarized in Table 1. None of the control subjects took antiplatelet agents, while 5 patients were treated with Acetylsalicylic Acid at the time of our evaluation. Moreover, 12 other patients and 4 controls were treated with statins, antihypertensive drugs and/or vitamin B supplementation. General Practitioners or other specialists had prescribed these drugs as part of primary vascular prevention strategy.

**Table 1:** Patients and Controls baseline characteristics.

	Patients	Controls	p-value
	N = 35	N = 35	χ2 test or Fisher exact test
	Average (standard deviation), or Number (%)	Average (standard deviation), or Number (%)	
Age (years)	51,1 (±9,3)	51,00 (±9,47)	0,96
Sex (M)	6 (17,1%)	6 (17,1%)	1,00
BMI	24,4 (±3,6)	24,2 (±4,1)	0,73
Smoke	11(31,4%)	8 (24,3%)	0,72
Alcohol abuse (≥2 unit/d)	0 (0,0%)	0 (0,0%)	—
Alcohol consumption (≤1unit/d)	15 (50,0%)	10 (30,3%)	0,14
Total Cholesterolemia (mg/dl)	214,7(±30,0)	198,6 (33,2%)	0,10
Creatinine (mg/dl)	0,79 (±0,11)	0,85 (±0,14)	0,09
Hypertension	7 (20.0%)	4 (11.4%)	0,29
Homocysteine (micromole/l)	11.45 (±3.08)	12.09 (±3.91)	0,86
Migraine	20 (57.1%)	13 (37.1%)	0,21
**Drugs**			
1. Antiplatelet agents	5 (14,3%)	0 (0%)	
2. Statins	4 (11,4%)	1 (2,9%)	
3. Antihypertensive	6 (17,1%)	2 (5,7%)	
4. Folate and B12 vitamins	2 (5,7%)	1 (2,9%)	
Time from MRI to visit (months)	10,2 (±9,5)	8,6 (±9,6)	0,57
**Fazekas score**			
5. Total	2,69 (±1,12)	0	—
6. Periventricular	1,12 (±0,67)	0	—
8. Deep white matter	1,64 (±0,70)	0	—

### Biomarkers

Patients and controls did not differ in any of the haemostatic biomarkers under study (patients vs. controls respectively): Fy (304,0 ± 69,1 vs. 304,0 ± 86,4; mg/dl; p = 0,99), tPA (6,3 ± 3,9 vs. 6,3 ± 2,3; ng/ml; p = 0,98), WVF-antigen (126,7 ± 43,9 vs. 123,9 ± 46,3; %; p = 0,80), WVF-activity (102,7 ± 33,1 vs. 100,5 ± 36,2; %; p = 0,80), PAI (7,5 ± 4,5 vs. 9,5 ± 6,6; ng/ml; p = 0,18), PAF-AH (18,5 ± 5,2 vs. 18,8 ± 2,9; nmol/min/ml; p = 0,84).

No difference was found also in CRP levels (2,3 ± 4,8 vs. 2,2 ± 3,4; mg/l; p = 0,92), IL10 (0,53 ± 1,1 vs. 0,27 ± 0,2; pg/ml; p = 0,42), Hcy (11,3 ± 3,0 vs. 12,2 ± 2,9; micromol/l; p = 0,24), NO levels (22,6 ± 13,9 vs. 22,7 ± 11,8; µM; p = 0,99).

We did not find any significant difference also in adhesion molecules plasma levels (patients vs. controls respectively): ICAM (217,7 ± 41,2 vs. 213,8 ± 49,5; ng/ml; p = 0,83), VCAM (561,7 ± 175,1 vs. 572,2 ± 208,7; ng/ml; p = 0,89), E-selectin (37,7 ± 16,3 vs. 31,7 ± 13,6; ng/ml; p = 0,33), P-selectin (19,9 ± 0,9 vs. 20,1 ± 1,0; ng/ml; p = 0,50).

We found significantly higher ADMA plasma levels in patients than in controls (70,44 ± 36,25 ng/ml vs. 46,58 ± 30,67 ng/ml; p = 0,004), and ADMA plasma levels presented a significant association with the presence of WMH, also after controlling for possible confounding factors in a multiple logistic regression model (Table 2).

**Table 2:** Multiple logistic regression model, testing ADMA levels, after controlling for hypertension (history of), Smoke habit (former or active smoker) and alcohol consumption (mild consumption, ≤1 unit/day); B, values for the logistic regression equation for predicting the dependent variable (patient status) from the different independent variables (log-odds units). S.E., Standard Error of B; O.R., Odds Ratio.

	B	S.E.	p-value	OR	95% C.I. Inferior	95% C.I. Superior
ADMA	0,027	0,009	0,002*	1,027	1,010	1,045
Hypertension	0,978	0,736	0,184	0,376	0,089	1,592
Smoke	0,412	0,617	0,504	0,662	0,198	2,219
Alcohol	1,190	0,582	0,041	0,304	0,097	0,951
COSTANT	0,267	0,944	0,777	—	—	—

**Figure 1:**
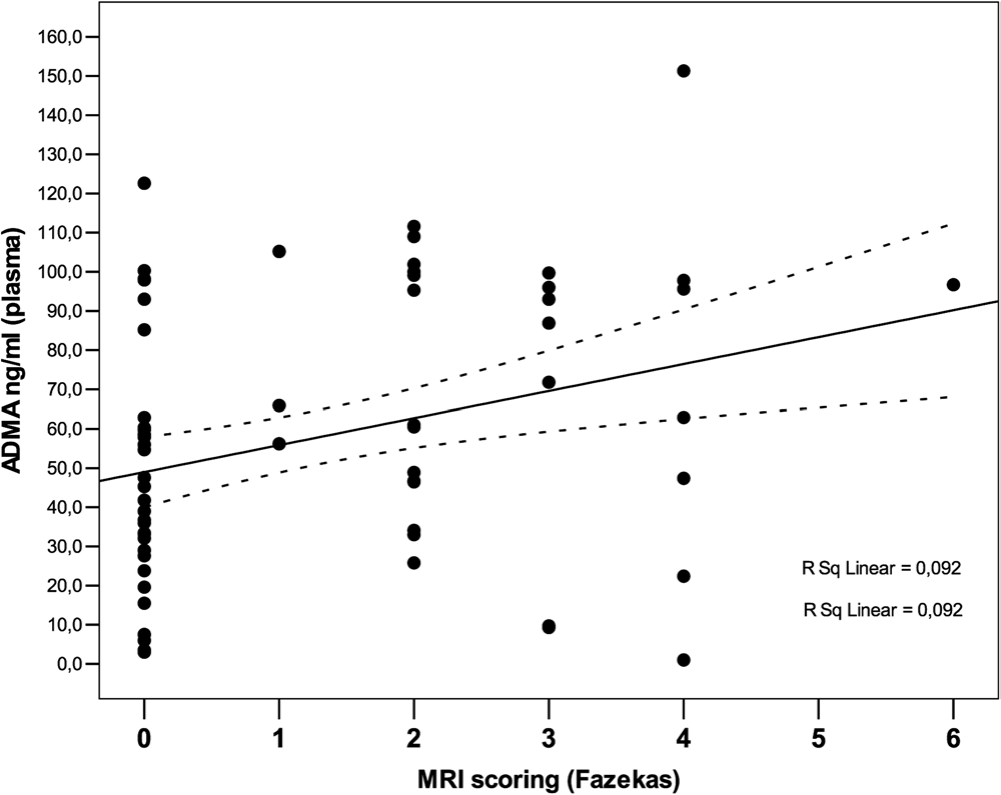
Overall correlation between ADMA levels and the Fazekas Score.

Moreover, ADMA levels did not correlate with age (r = 0,393; p = 0,11) or with homocysteine levels. In contrast, ADMA levels showed a positive significant, although limited, correlation with the total Fazekas score (r = 0,304; p = 0,01) (Fig. 1).

**Figure 2:**
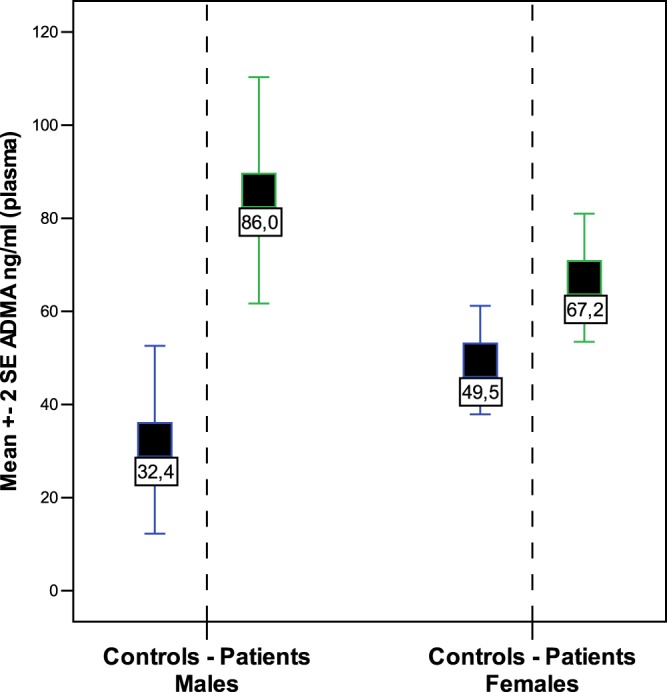
ADMA plasma levels in patient males and females versus the respective controls.

**Figure 3:**
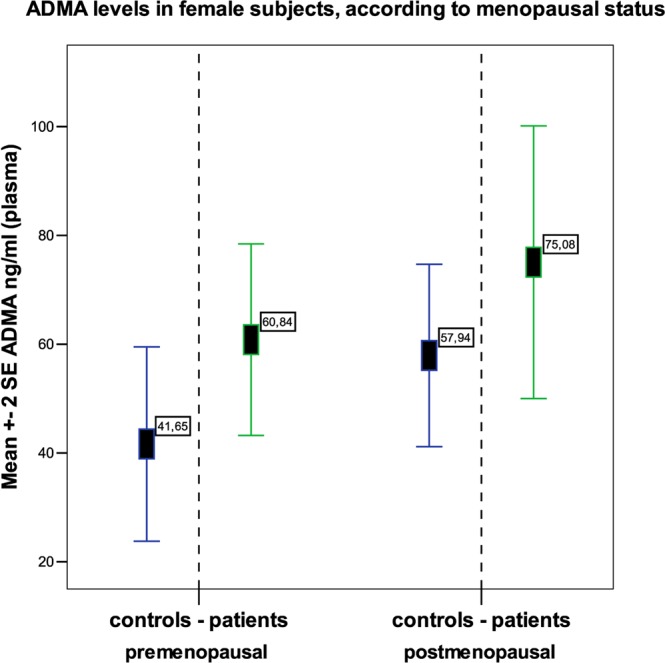
ADMA levels in plasma, in females, according to menopausal status. Data was obtained from 31 premenopausal (16 patients and 15 controls) and 26 postmenopausal women (12 patients and 14 controls).

**Figure 4:**
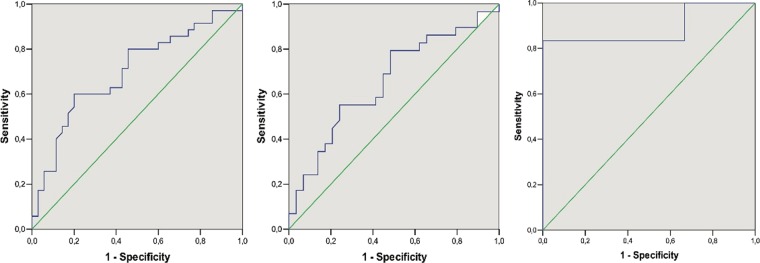
ROC cure analysis; from the left, in all (AUC = 0,70; CI 0,57-0,82 p = 0,004), female (AUC = 0,65; CI 0,51-0,78 p = 0,043) and male (AUC = 0,89; CI 0,67-1,10 p = 0,025) subjects respectively.

We found an important difference as regards to sex, since the difference in ADMA levels between patients and controls resulted more accentuated in males than in females (86,0 vs. 32,4; p = 0,007 and 67,2 vs. 49,5; p = 0,05 respectively; see Fig. 2). This different behaviour did not appear to be dependent on the menopausal status, since either in pre-menopausal and in post-menopausal women, ADMA levels tended to be higher in patients than in controls, although no significant difference was found (Fig. 3). ROC curve analysis shows an overall moderate sensitivity in discriminating patients and controls (AUC = 0,70; CI 0,57-0,82; p = 0,004); a cut-off of 46 ng/ml is associated with 80% sensitivity, but limited (54%) specificity. Separating males from females is possible to observe that in men the performance was more impressive (Fig. 4; AUC = 0,89; CI 0,67-1,10; p = 0,025); when setting the cut-off at 76 ng/ml, we obtained 83% sensitivity and 100% specificity. However, the small number of males should suggest considering with caution these result.

## Discussion

In this study we present a large laboratory assessment, which explore the different possible pathogenic mechanisms of WMH in cerebral small vessel damage: alterations in coagulation and fibrinolysis processes, inflammatory and autoimmune disorders, endothelial dysfunction. Several studies have already considered SVD, mainly in subjects displaying other vascular classical risk factors and including even strokes and Transient Ischemic Attacks or cardiovascular disease. The inclusion of all those confounders and conditions, which are known to have pathogenetic mechanisms (e.g. large vessels atherosclerosis, cardiac embolism, paradoxical embolism, vasospasm, vasculitis, etc.) very different from each other and from SVD in particular, appears in our opinion to be one of the main reasons of previous non-consistent results. In the same way, considering together all the SVD subtypes of lesions (WMH, perivascular enlarged spaces, brain atrophy, lacunas, microbleeds) may underlie different pathogenetic mechanism and might introduce further confounding factors. Among several, the few following evidences seem to substantiate this feeling: 1. Biomarkers evaluation in stroke setting proved to lead to different results according to stroke subtypes (e.g. cardioembolic vs. non-cardioembolic)^11^. 2. In a study analysing haemostatic markers of stroke recurrence (tPA, VWF and TAFI) their predictive value is consistent in coronary events but not in stroke^12^. 3. Some Authors suggest that blood biomarkers evaluation, interpretation and validation in the setting of cerebrovascular disease is highly dependent on the specific clinical setting^13^.

Consequently we tried to look at those patients, without clinical vascular disease (stroke in particular, but also heart disease and peripheral vessels disease), inflammatory brain disease (multiple sclerosis above all), who reach neurological evaluation for different complaints and in whom standard MRI examination reveals established features of SVD, such as WMH and PV hyperintesities.

Migraine is the most common condition associated with detection of WMH, but many other syndromes or single symptoms are represented: tension-type headache as well, vertigo and dizziness, mood disorders, non-specific cognitive complaints. The association between SVD and migraine is well known^14^ and almost 60% of our patients suffered from migraine. However, the few epidemiological studies do not provide specific data on the relationship between the occurrence of WMH and other SVD features on one side and migraine or other medical conditions on the other side^14^: consequently, we cannot exclude the possibility that a selection bias in MRI prescriptions could have led to a higher representation of migraneous subjects in our sample and in previous studies.

The panel of biomarkers we analysed was chosen after a review of the current literature (last update January 2016) on the most reliable biomarkers already tested. Poggesi *et al*.^6^ reported in 2015 a comprehensive overview of these biomarkers in the setting of SVD. Our results underline, in a subset of young (mean age 49,9 +/− 10.1), asymptomatic and with low burden of vascular risk factors (66,7% completely classical risk factor free) patients, the absence of evidence of latent inflammatory and autoimmune disease, as well as of coagulopathies. As far as the latter conditions are concerned, we actually did not looked in this study more deeply at other factors such as factor VII and VIII levels, or at the possible status of mutation carriers for Leiden, MTHFR, protrombine G20210A, etc. However, none of the subjects had a personal history of venous thrombosis or a family history of vascular disease. ADMA level seems to be the only significant marker in our panel, suggesting that endothelial dysfunction could play a pivotal role in asymptomatic SVD.

ADMA is one of the results of methylated proteins degradation. Differently to the others products, ADMA displays a unique inhibitor effect on endothelial nitric oxide synthase and leads to reduced levels of nitric oxide and consequently to possible endothelial dysfunction. Its plasma levels depend on some factors such as dietary habits, renal function and dimethylaminohydrolase (DDAH) activity^10^. Some studies have reported high ADMA concentrations in people with coronary and peripheral artery disease, chronic heart failure and pulmonary hypertension, pre-eclampsia and stroke^15–23^. ADMA levels are reported to be higher also in genetic conditions, such as CADASIL^24^. ADMA seems also to affect cerebral vascular compliance: in a double blind, placebo-controlled clinical study, Kielstein *et al*. demonstrated that i.v. injection of exogenous ADMA in 20 healthy volunteers, lead to a decrease of the cerebral blood flow compared to the placebo group, without modification of systemic blood pressure^25^. N.O. levels themselves are not reduced in our patients and no correlation is present between ADMA levels and NO levels: however they were determined in peripheral plasma and without controlling for several interfering factors, thus we suggest that this result was hard to interpret in the specific setting of our study and should be further explored. Altogether these data suggest that ADMA and nitric oxide pathway might be a common pathogenic point in different aetiologies of early phases of cerebral SVD. However, in the majority of the studies we reviewed in which higher ADMA levels were associated with SVD^26–30^, patients had stroke or at least several vascular risk factors and pro-atherosclerotic conditions, such as increased age, hypertension, hypercholesterolemia, diabetes mellitus, insulin insensitivity, hyper-homocysteinemia, etc. leading to an unclear relationship between ADMA levels and other factors.

The strength of our study is the highly selected subset of patients, but it has also some limitations, first of all the small sample size caused by the rigorous screening itself. Although unlikely because of absence of systemic signs or symptoms and familiar history, we didn’t perform genetic analysis to exclude hereditary diseases or heterozygous carriers status associated with sporadic SVD (e.g. CADASIL, CARASIL, MELAS, Fabry Disease, COL4A1/2 polymorphisms and other hereditary collagenopathies). We evaluated only WMH and no other features of SVD: patients had no lacunas or brain atrophy, but microbleeds and superficial siderosis, which could hint at brain vessels amyloidosis^31^ although unlikely in our young sample, are detected in MRI through GRE/SWI sequences, not performed as often as T2/Flair ones in routine MRI examinations.

## Conclusions and further developments

This study suggests an association between ADMA levels and WMH in early, asymptomatic SVD. The large panel of biomarkers investigated seems reliably to exclude inflammatory or coagulation/fibrinolysis disorders in these patients and reinforces the idea that endothelial dysfunction could have a pivotal role in early SVD pathogenesis. It will be important to perform serial MRI to follow-up progression of WMH and serial ADMA levels as well to assess inter and intra-assay variability of ADMA measurement. Future *in vitro* studies should assess the possible causal link between ADMA, endothelial dysfunction and SVD; further clinical studies should on the other hand focus on selected groups of patients.

If confirmed, the hypothetical pathogenetic role of ADMA pathway could open interesting perspectives, both from the diagnostic and the therapeutic point of view.

## Materials and Methods

### Study design

This is a prospective case-control study. Patients were enrolled consecutively in the neurological outpatient clinic of our Institution. Matching was used, for both age and gender, after each patient enrolment.

Our local ethic committee (Institutional Review Board of the Department of Medical Area, University of Udine) approved the study protocol (approval number 46/IRB/_gigli_16). Both patients and controls were enrolled after a written informed consent. Patients’ enrolment, handling and management of biological specimens and of subjects’ data were all performed in accordance with the relevant guidelines and regulations.

### Study population

Patients and controls were enrolled between January 2016 and February 2018. Patients attended our clinic for several complaints (see Suppl. Table) among which the most common were migraine, paraesthesia, dizziness, vertigo and subjective focal symptoms.

The inclusion criteria for patients were: (i) age between 18 and 65 years; (ii) white matter hyperintensities on T2-weighted/fluid attenuation inversion recovery (FLAIR) sequences at brain MRI performed with high field equipment (1,5 T), defined using STRIVE consensus^1^ and categorized by Fazekas score^32^. An expert neurologist who evaluated axial-FLAIR imagines of brain MRI calculated WMH scoring.

The exclusion criteria were: (i) two or more of the classical cerebrovascular risk factors (hypertension, diabetes, hypercholesterolemia, smoke), (ii) history of alcohol or drug abuse, (iii) extracranial carotid disease or panvasculopathy, (iv) history of ischemic heart disease, (v) thrombophilia (except hyper-homocysteinemia and heterozygous MTHFR gene mutation) (vi) symptomatic stroke, haemorrhage, transient ischemic attack or other neurologic disorders (dementia, epilepsy, multiple sclerosis, brain trauma, perinatal brain injury, neurodegenerative diseases), (vii) rheumatologic diseases (rheumatoid arthritis, vasculitis, connectivopathy), (viii) recent infection or surgery, (ix) malignancies, (x) chronic use of steroids, immunosuppressive or nonsteroidal anti-inflammatory drugs.

The control subjects were identified among people attending our Hospital for a brain MRI, who finally displayed a normal scanning and in which other neurological, vascular and chronic inflammatory diseases were excluded in a clinical interview.

All the clinical data were collected through a face-to-face interview with a neurologist, using a structured questionnaire which included: age, sex, weight and height, chronic pharmacological treatments, concomitant and previous diseases, hypercholesterolemia, history of hypertension, hyperhomocysteinemia, detection of patent foramen ovale, history of migraine, familiarity for cardio-cerebrovascular diseases, menopausal status. All patients and controls underwent to a general and neurological examination and to neck vessels ultrasound examination.

### Laboratory assessment

Both patients and controls underwent to the same blood drawing and analysis protocol. Blood samples were drawn between 08.00 a.m. and 11.00 a.m. (mean time 9:46 a.m. ± s.d. 52′ for patients and time 9:19 a.m. ± s.d. 52′ for controls), in order to limit as much as possible differences imputable to the circadian rhythm of biomarkers expression. Plasma or serum samples were kept on ice and aliquoted into 500 microL tubes to be stored at −80 °C until required.

An extended screening for thrombophilia and autoimmunity was conducted. In particular, anti-nuclear antibody (ANA), anti-extractable nuclear antigen (ENA), lupus anticoagulant (LAC), anti-cardiolipin IgG/IgM antibodies, anti-beta2 glycoprotein I IgG/IgM antibodies, anti-phosphatidylserine/prothrombin IgG/IgM antibodies, anti-thrombin III, protein C, protein S, fibrinogen (Fy), plasminogen activator inhibitor – 1 (PAI-1), tissue plasminogen activator (tPA), von Willebrand factor, homocysteine, C-reactive protein (CRP) were assessed using diagnostic assays.

In addition, we analysed levels of blood markers of inflammation and endothelial activation. Platelet activating factor-acetyl hydrolase (PAF-AH) and nitric oxide (NO) were measured by a colorimetric assay (Cayman Chemical – Michigan, US). Enzyme-linked immunoassays (ELISA) were used to measure levels of interleukin 10 (IL10) (Life Technologies, CA, US), E-selectin, P-selectin (R&D System, Minneapolis, US) and asymmetrical dimethyl-arginine (ADMA) (Casabio, Texas, US) following the manufacturers’ instructions. Serum concentration of vascular cell adhesion molecule-1 (VCAM-1) and intercellular adhesion molecule-1 (ICAM-1) were assayed by multiplex magnetic bead immunoassay (Bio-rad, California, US) according to the manufacturer’s instructions.

### Statistical analyses

Data are reported as mean ± standard deviation for continuous variables and frequency (%) for categorical variables. Case and control groups were compared using Student’s t test for continuous variables, while chi-square test were used for categorical data. Relationships between some variables were analysed by Pearson r correlational coefficient. The discriminative cut-off of significant variables values was evaluated by receiver-operating characteristic curves analysis and the predictive value was expressed as the area under the receiver operating characteristic curve.

All probability values are 2-tailed. A significance level of P < 0.05 was used for hypothesis testing.

Based upon the pilot analysis of 12 plasma samples tested for the extended screening specified in ‘Laboratory assessment’ and ADMA we determined that the standard deviation of ADMA levels (ng/ml) was 36,8 and the mean was 99.1. A sample of 25 patients provided 80% power to detect a one standard deviation difference in the normalized value of ADMA levels between patients and controls.

Statistical analyses were conducted with and SPSS 20 (SPSS Inc., Chicago USA).

## Supplementary information

supplementary information

## References

[1] Wardlaw JM (2013). Neuroimaging standards for research into small vessel disease and its contribution to ageing and neurodegeneration. Lancet Neurol..

[2] Wardlaw JM, Smith C, Dichgans M (2013). Mechanisms underlying sporadic cerebral SVD: insights from neuroimaging. Lancet Neurol..

[3] Benisty S (2009). Location of lacunar infarcts correlates with cognition in a sample of non disabled subjects with age-related white matter changes: the LADIS study. J Neurol Neurosurg Psychiatry..

[4] Pantoni L, Poggesi A, Inzitari D (2007). The relation between white matter lesions and cognition. Curr Opin Neurol..

[5] Pantoni L (2010). Cerebral small vessel disease: from pathogenesis and clinical characteristics to therapeutic challenges. Lancet Neurol..

[6] Poggesi A, Pasi M, Pescini F, Pantoni L, Inzitari D (2016). Circulating biologic markers of endothelial dysfunction in cerebral small vessel disease: a review. J Cerebral blood flow and Metab..

[7] Janes F (2013). Stroke Incidence and 30-day and six-month case fatality rates in Udine, Italy: a population-based prospective study. Int J Stroke..

[8] Cancelli I (2011). Incidence of transient ischemic attack and early stroke risk. Validation of the ABCD2 score in an Italian populatin-based study. Stroke..

[9] Han F (2018). Prevalence and risk factors of cerebral small vessel disease in a Chinese population-based sample. J Stroke..

[10] Sibal L, Agarwal SC, Home PD, Boger RH (2010). The Role of Asymmetric Dimethylarginine (ADMA) in Endothelial Dysfunction and Cardiovascular Disease. Curr Cardiol Rev..

[11] Montaner J (2008). Etiologic Diagnosis of Ischemic Stroke Subtypes With Plasma Biomarkers. Stroke..

[12] Pedersen A (2016). Haemostatic biomarkers are associated with long-term recurrent vascular events after ischaemic stroke. Thromb Haemost..

[13] Whiteley W, Tian Y, Jickling GC (2012). Blood biomarkers in stroke: research and clinical practice. Int J Stroke.

[14] Smith EE (2017). Prevention of stroke in patients with silent cerebrovascular disease. A scientific statement for healthcare professionals from the american heart association/america stroke association. Stroke..

[15] Valkonen VP (2001). Risk of acute coronary events and serum concentration of asymmetrical dimethylarginine. Lancet..

[16] Boger RH (1997). Biochemical evidence for impaired nitric oxide synthesis in patients with peripheral arterial occlusive disease. Circulation..

[17] Mittermayer F (2016). Asymmetric dimethylarginine predicts major adverse cardiovascular events in patients with advanced peripheral artery disease. Arterioscler Thromb Vasc Biol..

[18] Usui M, Matsuoka H, Miyazaki H, Ueda SOS, Imaizumi T (1998). Increased endogenous nitric oxide synthase inhibitor in patients with congestive heart failure. Life Sci..

[19] Gorenflo M, Zheng C, Werle E, Fiehn W, Ulmer HE (2001). Plasma levels of asymmetrical dimethyl-L-arginine in patients with congenital heart disease and pulmonary hypertension. J Cardiovasc Pharmacol..

[20] Pettersson A, Hedner T, Milsom I (1998). Increased circulating concentrations of asymmetric dimethyl arginine (ADMA), an endogenous inhibitor of nitric oxide synthesis, in preeclampsia. Acta Obstet Gynecol Scand..

[21] Yoo JH, Lee SC (2001). Elevated levels of plasma homocyst(e)ine and asymmetric dimethylarginine in elderly patients with stroke. Atherosclerosis..

[22] Lu TM, Ding YA, Lin SJ, Lee WS, Tai HC (2003). Plasma levels of asymmetrical dimethylarginine and adverse cardiovascular events after percutaneous coronary intervention. Eur Heart J..

[23] Meinitzer A (2007). Asymmetrical dimethylarginine independently predicts total and cardiovascular mortality in individuals with angiographic coronary artery disease (the Ludwigshafen Risk and Cardiovascular Health study). Clin Chem..

[24] Rufa A (2008). Plasma Levels of Asymmetric Dimethylarginine in Cerebral Autosomal Dominant Arteriopathy with Subcortical Infarct and Leukoencephalopathy. Cerebrovasc Dis..

[25] Kielstein JT (2006). ADMA Increases Arterial Stiffness and Decreases Cerebral Blood Flow in Humans. Stroke..

[26] Pikula A (2009). Association of plasma ADMA levels with MRI markers of vascular brain injury: Framingham offspring study. Stroke..

[27] Khan U, Hassan A, Vallance P, Markus HS (2007). Asymmetric dimethylarginine in cerebral small vessel disease. Stroke..

[28] Notsu Y (2009). Evaluation of asymmetric dimethylarginine and homocysteine in microangiopathy-related cerebral damage. Am J Hypertens..

[29] Gao Q (2015). S100B and ADMA in cerebral small vessel disease and cognitive dysfunction. J Neurol Sci..

[30] Guan J (2017). Analysis of risk factors in patients with leukoaraiosis. Medicine (Baltimore)..

[31] Linn J (2010). Prevalence of superficial siderosis in patients with cerebral amyloid angiopathy. Neurology..

[32] Fazekas F, Chawluk JB, Alavi A, Hurtig HI, Zimmerman RA (1987). MR signal abnormalities at 1.5 T in Alzheimer’s dementia and normal aging. AJR Am J Roentgenol.

