# Supplementary material for: ADMA as a possible marker of endothelial damage. A study in young asymptomatic patients with cerebral small vessel disease
Source: Sci Rep. 2019 Oct 2;9:14207. doi: 10.1038/s41598-019-50778-w (PMC6775279; doi:10.1038/s41598-019-50778-w)
Supplement: Supplementary file 1 — supplementary information [file 41598_2019_50778_MOESM1_ESM.pdf]

ADMA as a possible marker of endothelial damage. A study in young asymptomatic patients with cerebral small vessel disease.

Francesco Janes[1, #], Adriana Cifù[2], Maria Elena Pessa[1], Rossana Domenis[2], Gian Luigi Gigli[1], Nova Sanvilli[3], Annacarmen Nilo[1], Riccardo Garbo[1], Francesco Curcio[2], Roberta Giacomello[2], Martina Fabris[2], Mariarosaria Valente[1]

Affiliations

1 – Department of Neuroscience, S. Maria della Misericordia University Hospital, Udine, Italy.

2 – Department of Laboratory Medicine, S. Maria della Misericordia University Hospital, Udine, Italy.

3 – Department of Medical Area (DAME), University of Udine, Udine, Italy

#Address correspondence to:
Francesco Janes, MD, Ph.D.
Department of Neuroscience, S. Maria della Misericordia University Hospital
University of Udine Medical School
15th S. Maria della Misericordia Square, Udine (UD), 33100
Phone: +39 – 349 - 8667162
francesco.janes@gmail.com
francesco.janes@asuiud.sanita.fvg.it

Supplementary Table

Specific clinical indications for Brain MRI scanning in patients and control subjects.

	**Patients**	**Controls**
**Migraine (with aura) ***	14 (2)	7(3)
**Paresthesias**	5	1
**Focal subjective symptoms**	4	-
**Vertigo and Dizziness**	3	8
**Hypoacusia**	-	7
**Isolated Palpebral Ptosis (MG excluded)**	-	3
**Atypical Facial Neuralgias**	2	-
**Diffuse subjective symptoms**	2	2
**Tension-type Headache**	1	2
**Tremor (non ED, non PD, non dystonic features)**	-	2
**Tinnitus**	1	1
**Idiopathic facial palsy**	1	1
**Emifacial spasm**	1	-
**Aspecific memory complaints**	1	-
**Syncope**	-	1

* Overall 20 patients and 13 controls suffered from migraine but MRI was performed according to other incidental complaints; ED, Essential Tremor; PD, Parkinson disease; MG, Myasthenia Gravis.
